# Assessment of the Polyphenol Indices and Antioxidant Capacity for Beers and Wines Using a Tyrosinase-Based Biosensor Prepared by Sinusoidal Current Method

**DOI:** 10.3390/s19010066

**Published:** 2018-12-25

**Authors:** Juan José García-Guzmán, David López-Iglesias, Laura Cubillana-Aguilera, Cecilia Lete, Stelian Lupu, José María Palacios-Santander, Dolores Bellido-Milla

**Affiliations:** 1 Institute of Research on Electron Microscopy and Materials (IMEYMAT), Department of Analytical Chemistry, Faculty of Sciences, Campus de Excelencia Internacional del Mar (CEIMAR), University of Cadiz, Polígono del Río San Pedro, S/N. 11510 Puerto Real, 11001 Cadiz, Spain; juanjo.garciaguzman@uca.es (J.J.G.-G.); david.lopeziglesias@uca.es (D.L.-I.); laura.cubillana@uca.es (L.C.-A.); 2 Institute of Physical Chemistry “Ilie Murgulescu” of the Romanian Academy, 202 Splaiul Independentei, 060021 Bucharest, Romania; cecilia_lete@yahoo.com; 3 Department of Analytical Chemistry and Environmental Engineering, Faculty of Applied Chemistry and Materials Science, University Politehnica of Bucharest, 1-7 Gh. Polizu Street, 011061 Bucharest, Romania

**Keywords:** sonogel–carbon based biosensor, sinusoidal current method, polyphenol index, antioxidant capacity, beer and wine

## Abstract

The application of a novel Poly(3,4-ethylenedioxythiophene)-Tyrosinase/Sonogel-Carbon electrode (PEDOT-Tyr/SNGC) biosensor to beers and wines analysis is proposed. This biosensor implies a new Sinusoidal Current (SC) electrodeposition method to immobilize the enzyme generating a nanostructure surface. The biosensors were characterized electrochemically, employing cyclic voltammetry and electrochemical impedance spectroscopy. Sensitivity, limit of detection, and correlation coefficients of the linear fitting were 2.40 × 10^−4^ µA·µM^−1^, 4.33 µM, and R^2^ = 0.9987, respectively. Caffeic acid is used as the reference polyphenol. A sampling of nine beers (four lager, three stout, and two non-alcoholic beers), and four wines (three red and one white wine) purchased in a local store was performed. The Polyphenol indeces for beers and wines have been assessed using the proposed biosensor, and the obtained values are in agreement with the literature data. Antioxidant properties of the samples using the 2,2′-azino-bis(3-ethylbenzothiazoline-6-sulphonic acid (ABTS) radical spectrophotometric method were also evaluated. The correlation between the polyphenol index and the antioxidant capacity was obtained for beers and wines.

## 1. Introduction

According to International Organisms, beers and wines are two of the most consumed beverages in the world; the production during 2016 was 1.96 × 10^11^ and 2.59 × 10^10^ L, respectively [1,2]. Despite the cultural and traditional factors related to these drinks, it has been previously demonstrated that these beverages provide health benefits when their consumption is moderated, mainly, due to their antioxidant capacity [3,4]. Antioxidant capacity is involved in the scavenging of reactive oxygen species (ROS) which are the main culprits responsible for a great number of diseases, such as cellular aging, mutations, and even cancer [5]. Within the great number of compounds which can be found in beers and wines, polyphenols have an important role in the above-mentioned antioxidant capacity [6,7,8,9,10,11,12]. Furthermore, the role of these compounds has been demonstrated in human health, such as anti-inflammatory and cardioprotective roles, among others [13]. In spite of the evident importance of these compounds as antioxidants, there is no reliable reference methodology to determine that the polyphenols are related to the antioxidant capacity in beverages. Powerful techniques, such as high-performance liquid chromatography (HPLC) or HPLC coupled to mass spectrometry can be used to determine the individual polyphenols present in beverages [14,15]. However, they are expensive techniques which demand qualified personnel and long analysis times relative to other techniques. Besides, the single determination of polyphenols lacks any practical result in many cases. Other techniques employ a different approach, determining a collective group of polyphenols. In this case, spectrophotometric and electroanalytical methods are frequently used. One of the most used as a reference method is the Folin–Ciocalteau assay [16], a spectrophotometric method which uses an oxidant mixture that reacts with the reducing substances in the sample. Therefore, the results obtained with this method are more related to the reducing capacity than the polyphenol content [7]. In the special case of red wine, the reducing sugar concentrations are above eight times the total concentration of polyphenols [17], which may imply an overestimation of the polyphenol content. Another approach typically used to measure the antioxidant capacity of the sample is based on the addition of a radical which simulates those ones formed in the sample. The exogenous radicals are formed and native sample antioxidants scavenge them; this process can be monitored by spectrophotometric measurements. Different radicals species have been used to measure the antioxidant capacity in beverages, such as (2,2′-azino-bis(3-ethylbenzothiazoline-6-sulphonic acid)) ABTS [18], (Dimethyl-4-phenylenediamine) DMPD [19], and (2,2-diphenyl-1-picrylhydrazyl) DPPH [20]. ABTS could be more suitable since it implies a more drastic radical similar to the radicals naturally formed in this kind of samples. The decrease in the absorbance is related to the antioxidant capacity of the sample [9].

Electroanalytical techniques have been pointed out as a suitable alternative, taking into account several interesting features, such as simple instrumentation, sensitivity, quick response, no sample treatment, low cost, compact size, and the possibility of in situ and online analysis remarking the special interest for wineries. Moreover, the combined use of enzymes with electroanalytical techniques provides adequate selectivity for the polyphenol index determination. In this context, enzymatic biosensors have been widely studied to determine polyphenols in beverages [20,21,22,23,24,25,26,27]. Tyrosinase and laccase enzymes have been frequently used due to their associated substrates, o-diphenols and monophenols, which have been previously related to the antioxidant capacity of beverages [7,9]. On the other hand, biosensors have been improved using many different approaches in the last decades. A typical improvement is the deposition of a conductive layer onto the surface of the electrode, thereby enhancing the electrochemical properties of the material; additionally, this layer adequately supports the immobilization of the enzyme [28,29,30,31,32,33]. The electrodeposition of conducting polymers (CPs) for the development biosensors has usually been achieved by classical potentiostatic and/or galvanostatic methods. Recently, our group developed a new methodology based on the superimposition of sinusoidal voltages (SV) for electrodeposition of the CP layer and/or immobilizing the enzyme simultaneously [28,29,30,31]. This approach has generated an improvement in the enzymatic activity due to the high compatibility of these materials. A novel methodology based on the use of sinusoidal currents (SC) has also been applied in the successful development of electrochemical nanostructured biosensors for the determination of catechol and dopamine in synthetic samples, as well as dopamine in a commercial drug [34]. This simple method could be easily applied in food laboratories or in wineries or breweries.

In this work, the SC methodology is used to fabricate the biosensor, PEDOT/Tyr-SNGC. This biosensor is applied to determine the polyphenol index in beer and wine samples which were commercially available to consumers. The polyphenol index was related to the antioxidant capacity of the samples by employing the spectrophotometric ABTS assay.

## 2. Materials and Methods

### 2.1. Reagents and Chemicals

Hydrochloric acid (Panreac, Barcelona, Spain), methyltrimetoxisilane (MTMOS) (Merck, Darmstadt, Germany), graphite powder UF (Alfa Aesar, Karlsruhe, Germany), sulfuric acid (Merck), 3,4-ethylenedioxythiophene (Sigma-Aldrich, Steinheim, Germany), potassium dihydrogen phosphate (Merck), di-potassium hydrogen phosphate (Panreac, Barcelona, Spain), Tyrosinase (E.C. 1.14.18.1, from mushroom, 3610 units/mg solid, Sigma-Aldrich, Steinheim, Germany), potassium hexacyanoferrate (II) (Sigma-Aldrich, Steinheim, Germany), potassium hexacyanoferrate (III) (Sigma-Aldrich, Steinheim, Germany), caffeic acid (Sigma-Aldrich, Steinheim, Germany), hexan-1-ol (Panreac, Barcelona, Spain), 2,2′-Azino-bis(3-ethylbenzthiazoline-6-sulphonic acid) diammonium salt (ABTS) (Sigma-Aldrich, Steinheim, Germany) and potassium peroxydisulphate (Panreac, Barcelona, Spain) were used as received, without further purification.

All aqueous solutions were prepared with nanopure water which was obtained by passing twice-distilled water through a Milli-Q system (18 MΩ cm, Millipore, Bedford, MA, USA).

### 2.2. Real Samples

A sampling of four lager beers (Steinburg, Mahou, San Miguel, Carlsberg), three stout beers (Guiness, Cusqueña, Negra) and two non-alcoholic beers (Mahou sin, Buckler 0.0) was performed. Three red wines (Cariñena Reserva, Cariñena Crianza and Rioja Comportillo) and a white wine (Tierra Blanca) were also selected. All samples, wines and beers, were commercially available and were obtained in a local store.

### 2.3. Electrochemical Measurements

All electrochemical measurements were performed using an Autolab potentiostat/galvanostat 302 N (Ecochemie, The Netherlands) equipped with FRA2 module, in a three-electrode configuration. The working electrodes were made of Sonogel-Carbon electrodes (SNGC), with an inner diameter of 1.15 mm, prepared according to a previously published procedure [35,36]. The reference electrode was a Ag/AgCl/KCl (3 M) electrode (Metrohm, Herisau, Suiza). A glassy carbon rod (Metrohm, Herisau, Suiza) was used as the counter electrode. Before being used, the working SNGC electrodes were electrochemically activated in 0.1 M H_2_SO_4_ aqueous solution by two polarization steps at −0.7 V for 10 s, and at +1.8 V for 10 s, respectively. This electrochemical procedure was repeated eight times. The electrochemical characterization of the working electrodes was achieved using the electrochemical impedance spectroscopy technique by recording the spectra with the FRA2 module in an aqueous solution containing 5 mM K_4_Fe(CN)_6_/K_3_Fe(CN)_6_, and 1 M KCl, in the frequency range from 10 kHz to 0.05 Hz, with 5 mV amplitude of the sine wave, at a bias potential of 0.22 V (the potential of the redox couple). The quantitation of the analytes was performed in an air-saturated buffered aqueous solution.

### 2.4. Electrodeposition Procedure of PEDOT-Enzyme Layers

The preparation of PEDOT-Tyr/SNGC biosensors was carried out in an aqueous solution, with the following optimized composition [34]: 0.01 M 3,4-ethylenedioxythiophene (EDOT), 2 mg/mL Tyr, and 0.05 M phosphate buffer solution (PBS) of pH 7, by applying the following electrochemical procedure: Sinusoidal currents (SC) of single sine wave type with fixed frequency of 100 mHz and amplitude of 1.0 μA were superimposed onto a direct current (d.c.) of 4 μA. An electrodeposition time of 300 s was applied to prepare the PEDOT-Tyr coatings. These biosensors were washed with Milli-Q water and used in the analytical applications. After the measurements, the biosensors were kept in a refrigerator at 4 °C.

### 2.5. Electroanalytical Measurements

The analytical quantitation of caffeic acid, as the reference polyphenol, was performed in air-saturated aqueous buffer solutions (pH 7) at room temperature by using chronoamperometry at a working detection potential of 0.17 V, stirring at 150 rpm approx., by the standard addition method. The polyphenol index (PI) of the real samples was expressed as caffeic acid content and measured similarly in amperometric detection mode by a standard addition method. Beer samples were degassed adding several drops of hexan-1-ol to 25 mL of beer. Red wines were diluted five times with Milli-Q water. All the analytical measurements were carried out in triplicate.

### 2.6. Spectrophotometric Measurements

ABTS was dissolved in water to a concentration of 7 mM, the ABTS radical cation (ABTS^+^) was obtained by reacting with 2.45 mM potassium peroxydisulphate and allowing the mixture to stand in darkness at room temperature for 16–20 h before use. A quantity of 0.5 mL was taken and diluted to 25 mL with Milli-Q water. In the case of wines, 15 µL of Milli-Q water was added to 2.5 mL of ABTS^+^ and the absorbance was measured at 734 nm. After that, 15 µL of wine was added to 2.5 mL of ABTS^+^ and the absorbance was measured at the same wavelength. The decrease in absorbance was recorded in this case. On the other hand, 100 µL of beers was used to perform the beer analysis using the same procedure.

## 3. Results

### 3.1. Electrochemical Characterization of PEDOT-Tyr/SNGC Biosensor

In the present work, an electrochemical characterization was carried out with the aim of ensuring the successful preparation of the proposed biosensor. The morphological studies were assessed in a previous paper [34].

**Figure 1:**
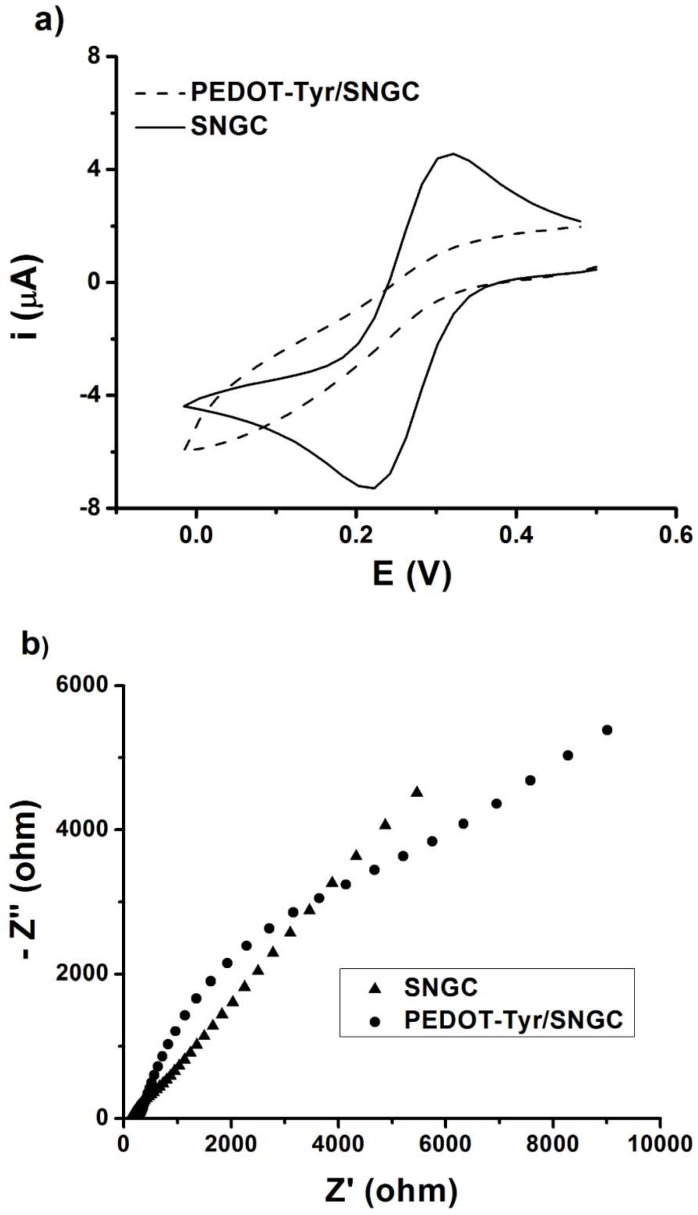
Electrochemical characterization of poly(3,4-ethylenedioxythiophene)-Tyrosinase/Sonogel-Carbon (PEDOT-Tyr/SNGC) and Sonogel-Carbon (SNGC) electrodes in an aqueous solution containing 5 mM K_3_Fe(CN)_6_, and 1 M KCl. (**a**) Cyclic voltammetry, (**b**) electrochemical impedance spectroscopy (EIS) spectra recorded in an aqueous solution containing 5 mM K_3_Fe(CN)_6_/K_4_Fe(CN)_6_, and 1 M KCl.

Firstly, cyclic voltammetry was performed to study the electrochemical behavior of the biosensor. Potassium hexacyanoferrate (III) was used as the redox probe in these studies. The cyclic voltammograms were recorded between the cathodic and anodic potential limits of 0.0 and 0.5 V, respectively, at the potential scan rate of 50 mV·s^−1^. In order to check the successful electrodeposition of the PEDOT-Tyr coating, the cyclic voltammograms were also recorded at a bare SNGC electrode for comparison. In addition, these electrodes were also characterized by using electrochemical impedance spectroscopy (EIS) in an aqueous solution containing both forms of the redox couple in equal concentrations. The results from these measurements are shown in Figure 1a. On the other hand, the impedance response is presented in Figure 1b.

### 3.2. Kinetic Constants and Analytical Performances of the PEDOT-Tyr/SNGC Biosensor

**Figure 2:**
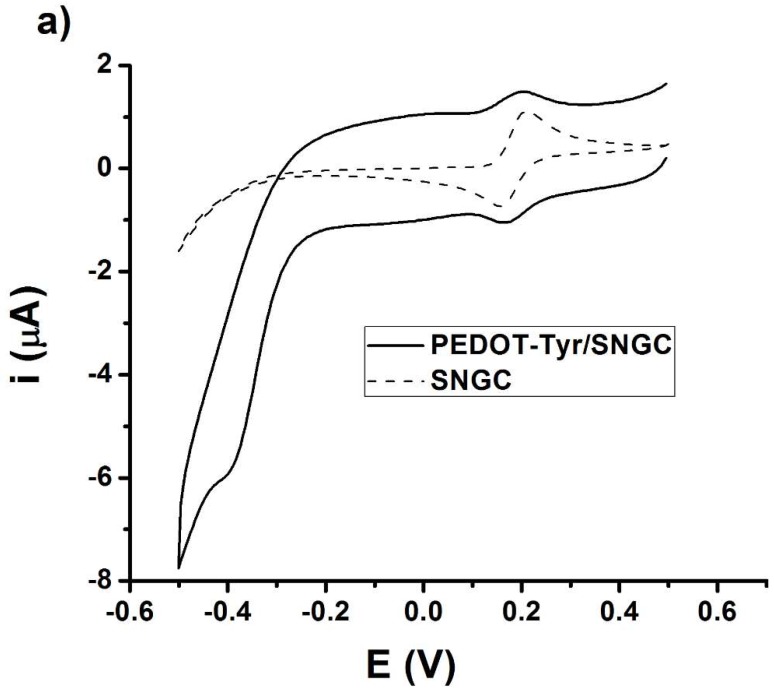

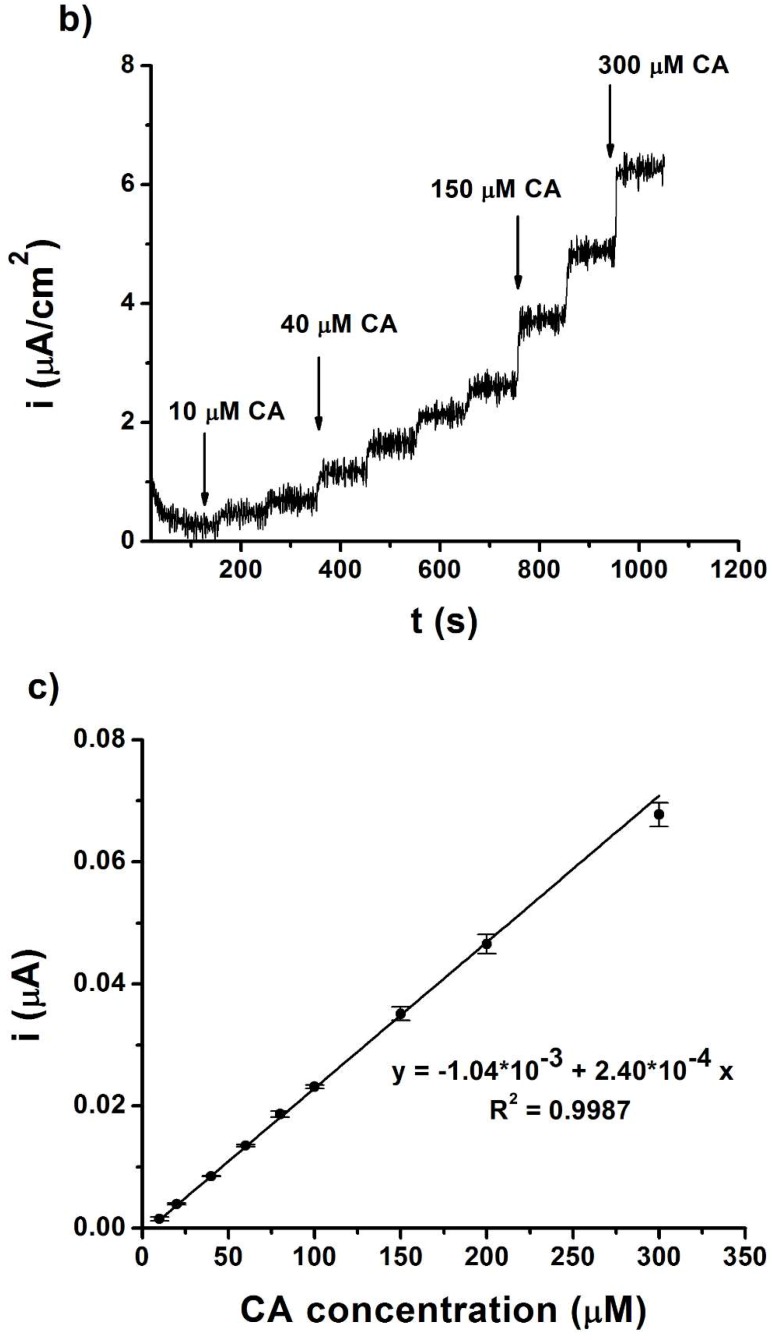
(**a**) Cyclic voltammograms recorded at SNGC and PEDOT-Tyr-SNGC electrodes in a 0.1 M phosphate buffer aqueous solution containing 300 µM caffeic acid, at 50 mV/s potential scan rate; (**b**) Chronoamperogram corresponding to caffeic acid additions (10, 20, 40, 60, 80, 100, 150, 200, and 300 µM) at the PEDOT-Tyr/SNGC biosensor; (**c**) calibration plot with error bars expressed as standard deviation.

The electrochemical behavior of caffeic acid was investigated in an aqueous buffered solution at both SNGC and PEDOT-Tyr/SNGC electrodes by means of a cyclic voltammetry method, see Figure 2a. A reversible wave with a redox potential of ca. 0.2 V can be observed for both electrodes and this wave is ascribed to the reversible electrochemical reduction–oxidation of caffeic acid. The PEDOT-Tyr/SNGC biosensor displays a second cathodic wave with peak potential at ca. −0.38 V, characteristic of the reduction of the quinone derivative formed in the enzymatic reaction. This wave is absent in the case of an unmodified SNGC electrode and this attests to the proper immobilization of the tyrosinase enzyme within the PEDOT layer.

The chronoamperometry method was selected to determine caffeic acid (CA) because of its higher sensitivity, and 0.17 V was chosen as the optimum working detection potential value. This working detection potential value was selected because it is equal to the open circuit potential value and less transient, non-faradaic currents and interferences are expected. The kinetic parameters, the Michaelis–Menten constant (K_M_), and the maximum rate under saturated substrate expressed as the maximum current (I_max_) were evaluated. The obtained K_M_ and I_max_ values were 178.72 µM and 2.31 × 10^−2^ µA, respectively. The analytical response of the PEDOT-Tyr/SNGC biosensor toward caffeic acid was investigated in aqueous buffered solutions by means of the chronoamperometry method, see Figure 2b.

Figure 2b shows the chronoamperogram obtained by the addition of different concentrations of caffeic acid (10–300 µM). The corresponding calibration plot, see Figure 2c, was built employing the increase of current intensity for each CA addition. Limits of detection, quantitation, and sensitivity for the PEDOT-Tyr/SNGC biosensor toward caffeic acid were 4.33 µM, 14.43 µM, and 2.40 × 10^−4^ µA·µM^−1^, respectively. The limits of detection (LD) and quantitation (LQ) were assessed using the IUPAC criteria: 3 s/m and 10 s/m, respectively, where *s* is the standard error of the linear regression intercept and m is the slope of the calibration plot.

The repeatability of the measurements was evaluated by constructing successive calibration plots for CA in a linear range from 10–300 µM, obtaining a relative standard deviation (RSD) value of 2.67%. Moreover, the operational working time of one single biosensor was tested by carrying out several calibration plots for CA in the same abovementioned concentration range. Each electrode was kept in the refrigerator at 4 °C after the daily measurements. No significant differences were observed for the biosensor signal over a period of at least 10 days.

### 3.3. Applications of PEDOT-Tyr/SNGC Biosensor for the Determination of the Polyphenol Index in Beers and Wines

The next step was the application of the developed biosensor to beer and wine samples. For this purpose, a standard addition method was employed to determine the polyphenol index by using, in both cases, caffeic acid as the reference polyphenol. Chronoamperometry was chosen as the electroanalytical technique to determine this index due to its higher sensitivity and the satisfactory results obtained in the previous studies [7,35,36,37]. The measurements were carried out by adding the sample (beers or wines) to the electrochemical cell containing buffer solution and, after that, different aliquots of the reference standard (CA) were also added. Additionally, this procedure can be applied without pretreatment of the samples; only a dilution with Milli-Q water was required (in the case of red wines), compared to laboratory-based analytical methodologies that involve sample preparations steps.

**Figure 3:**
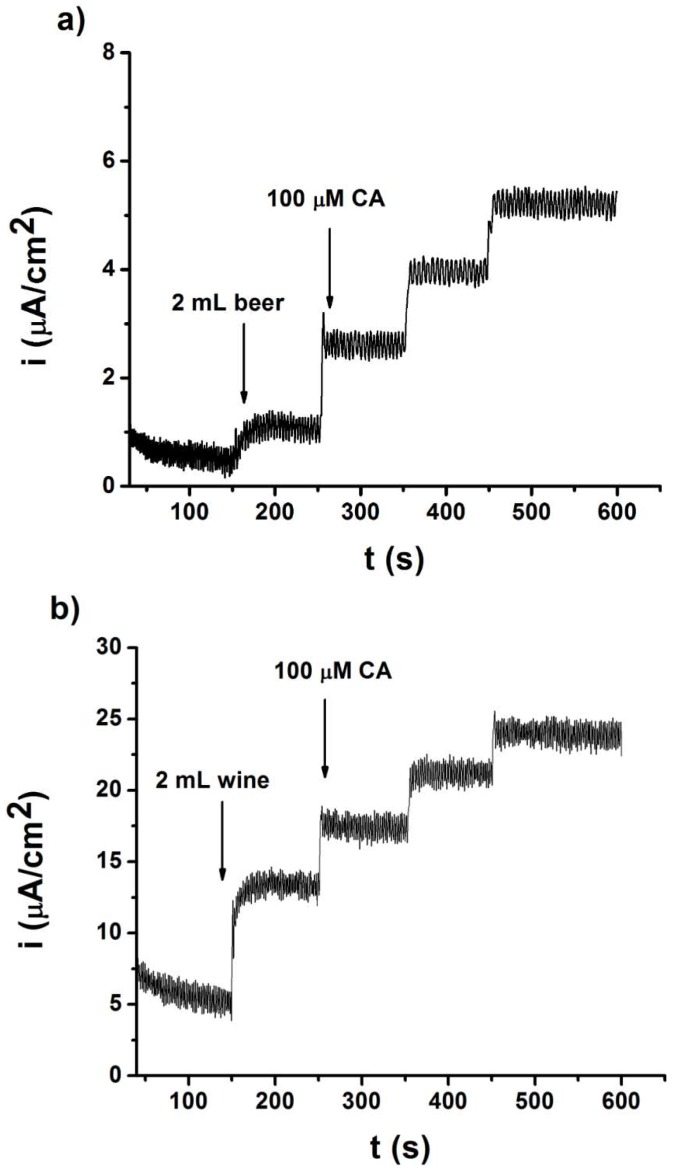
(**a**) Chronoamperogram recorded with the PEDOT-Tyr/SNGC biosensor for beer analysis using the standard addition method (100, 200, and 300 µM CA additions). (**b**) Chronoamperogram recorded at the PEDOT-Tyr/SNGC biosensor for red wine analysis using the standard addition method for various CA additions (100, 200, and 300 µM).

The biosensor was applied in the analysis of nine beers: Four lager beers, three stout beers, and two beers without alcohol. These types of beers were selected because of their highest level of consumption. All chronoamperometric measurements were performed at a working detection potential of 0.17 V. Figure 3a shows the chronoamperogram recorded for the direct analysis of a lager beer by a standard addition method. As observed, a linear increase of the current response was obtained when the beer and aliquots of CA were added. The standard addition equation was y (µA cm^−2^) = 1.38 × 10^−2^·x (µM) + 6.80 × 10^−1^ with a linear fitting correlation coefficient of 0.997.

On the other hand, the PEDOT-Tyr/SNGC biosensor was used to determine the polyphenols index using the standard addition method for a white wine and three red wines. The red wine group included a “Crianza” wine (at least 24 months in an oak cask) and “Reserva” wine (at least 36 months in an oak cask). Figure 3b shows the chronoamperogram recorded for a red wine using a dilution factor 1:5 and spiked with several aliquots of the CA standard solution. The intensity of the current increases linearly with the CA concentrations and the standard addition fitting equation was y (µA cm^−2^) = 3.56 × 10^−2^·x(µM) + 8.30 with a linear fitting correlation coefficient of 0.991.

The polyphenol index and RSD % for the PEDOT-Tyr/SNGC biosensor used in the analysis of beers and wines samples is summarized in Table 1. All results were obtained in triplicate. Good RSD (%) values were obtained for all assays, almost all of them lower than 5%. In addition, repeatability of the response was also studied using three independent biosensors with the same beer sample; the value of reproducibility (RSD) was found to be 7.98%.

The analysis of several samples of wines and beers was successfully achieved using the proposed amperometric biosensor. Furthermore, the analytical performance in terms of linear response range, limit of detection, and repeatability of the PEDOT-Tyr/SNGC biosensor is comparable to other biosensors based on polyphenol oxidases or peroxidases reported previously in the literature, see Table 2.

### 3.4. Determination of Antioxidant Capacity in Beers and Wines Using the Spectrophotometric ABTS Assay

**Figure 4:**
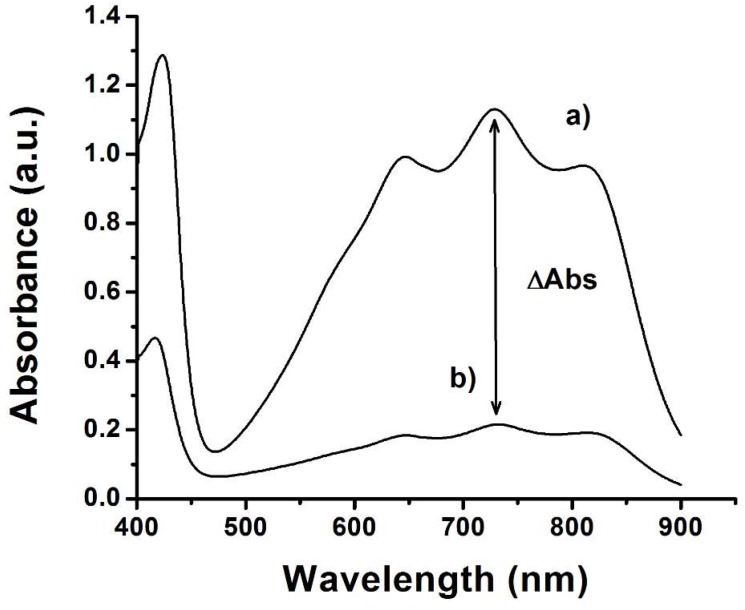
Spectrophotometric measurements obtained using an ABTS radical assay: (**a**) ABTS with addition of the blank and (**b**) ABTS with a lager beer sample added.

Figure 4 shows two different spectra for a lager beer: (a) correspond with the ABTS^+^ without addition of the sample and (b) correspond with the addition of the sample.

The addition of the sample to ABTS radical causes a decrease in the absorbance signal around 730 nm. The variation of the absorbance (ΔABTS) is related to the antioxidant capacity of the sample [9]. The results obtained are summarized in Table 1. All the assays were carried out in triplicate.

## 4. Discussion

As it can be observed in Figure 1a, there is a decrease of both the cathodic and anodic peak currents of the redox probe recorded at the PEDOT-Tyr/SNGC electrode due to the presence of the immobilized enzyme, in comparison with the unmodified SNGC electrode. The enzyme is increasing the resistance of the PEDOT-Tyr coating; thus, the electron transfer at the electrode/solution interface is hindered and causes a decrease in the peak currents.

On the other hand, according to Figure 1b, the unmodified electrode has a typical response based on a straight line forming a 45° degree angle with the X-axis, related to the Warburg impedance, showing classical behavior of a bare electrode. However, the EIS spectrum for PEDOT-Tyr/SNGC is characterized by an increase of the charge transfer resistance (R_CT_). The determined values of R_CT_ were 1372 Ω in the case of the SNGC electrode and 9093 Ω for the PEDOT-Tyr/SNGC modified electrode, respectively. The higher resistance is related to the deposition of the polymer-enzyme layer onto the surface of the electrode. All these results confirmed the successful electrodeposition of the modifying layer.

Regarding the values obtained for K_M_ (178.72 µM) and I_max_ (2.31 × 10^−2^ µA), the low value of 178.72 µM indicated a great affinity of the enzyme for the substrate. Consequently, this can be translated into a very suitable environment for the enzyme immobilized inside the polymer matrix which did not change the activity of this enzyme

The results regarding the most important quality analytical parameters, such as limits of detection and quantitation and sensitivity for the PEDOT-Tyr/SNGC biosensor toward caffeic acid are in good agreement with those published in the literature [37,38,39,41].

Concerning the analysis of beer samples shown in Table 1, the highest polyphenol indices were obtained for stout beers. The results were in agreement with those reported in the literature [9]. With the aim to differentiate the types of beers, an analysis of the variance with a significance level of 95% was performed. The F ratio was 43.88 and *p*-value was 0. These results indicate that there are significant differences among beers. The multiple range tests confirmed that stout beers have a higher polyphenol content than other beers. These studies demonstrated the potentiality of the biosensor for the discrimination of the polyphenol content between lager and stout beers.

Furthermore, as is also evident from Table 1, red wines provide a higher polyphenol index in comparison with white wines and beer samples, as expected. The polyphenol content is strongly related to different factors such as grape variety, environmental factors, and maduration in wood. Moreover, wine-making techniques are also a critical factor in the polyphenol content in wines and they are also highly related to the quality of these kinds of beverages. In addition, red wines have a much higher content of flavonoids, anthocyanins, and flavanols, which provides the typical color and astringency [44]. On the other hand, flavonoids present in white wine appear at very low concentration levels. Excellent repeatability was found, with a RSD value lower than 5%. Two red wines of the same trademark with different aging in oak cask were studied, showing an increase in the polyphenol index for “Reserva” wine; this can be attributed to the polyphenols incorporated from the oak cask during the aging.

Taking into account the results exposed in Table 2, the use of SC methodology for biosensor fabrication involves an easier and faster procedure (only five min of deposition time are required) with a minor amount of reagents used, in comparison with those that involve a drop-casting method.

Finally, the applications of the tyrosinase amperometric biosensor obtained via a novel sinusoidal current method have been achieved. The polyphenols index for several types of wines and beers has been successfully assessed using a selective device. The biosensor displayed good analytical performance, such as limits of detection and quantitation, a linear response range, reproducibility, stability, precision, and accuracy, with caffeic acid as the polyphenol reference. Finally, it is important to highlight the following advantages of the proposed biosensor: the simplicity; the enzyme and the polymer deposition is done in a single step simultaneously and in few minutes, and the rapidity; the response is obtained instantly and at a low cost, which is lower than 2 euros per biosensor (estimation according to the cost of fabrication and reagents). For all these above-mentioned reasons, this biosensor can be proposed as a tool for polyphenol index monitoring during the elaboration of these beverages.

## 5. Conclusions

The applications of a tyrosinase-based amperometric biosensor obtained via a novel sinusoidal current method for the determination of polyphenols index in wines and beers have been achieved. The biosensor displayed good analytical performance, such as limits of detection and quantitation, linear response range, reproducibility, stability, precision, and accuracy, with caffeic acid as polyphenol reference. The polyphenols index for several types of wines and beers has been successfully assessed using a selective analytical device. The developed PEDOT-Tyr/SNGC biosensor showed outstanding properties in the assessment of the polyphenolic composition of beers and wines offering useful information about the antioxidant capacity of these beverages.

## Figures and Tables

**Table 1:** Polyphenol index (PI) and antioxidant capacity (ΔABTS) values for beers and wines using PEDOT-Tyr/SNGC biosensor and spectrophotometric 2,2′-azino-bis(3-ethylbenzothiazoline-6-sulphonic acid (ABTS) assay. RSD = relative standard deviation.

Samples	PI (µM) Referred as CA	RSD (%)	ΔABTS	RSD (%)
Mahou (lager)	55.9	4.7	0.914	0.67
Steinburg (lager)	39.0	3.6	0.527	0.82
Carlsberg (lager)	45.6	6.00	1.065	0.81
San Miguel (lager)	33.9	11.5	0.890	1.15
Guinnes (stout)	68.6	3.9	1.406	0.61
Cusqueña (stout)	97.9	1.6	1.711	0.8
Negra (stout)	104.4	2.7	1.332	1.22
Mahou (non-alcoholic)	45.2	4.1	0.697	1.13
Buckler 0.0 (non-alcoholic)	54.3	6.8	0.738	0.13
Cariñena Reserva (red wine)	1186.3	1.3	1.943	0.95
Cariñena Crianza (red wine)	936.0	0.2	1.764	1.2
Rioja Comportillo Reserva (red wine)	1181.5	3.8	1.625	0.55
Tierra blanca (white wine)	130.0	0.7	0.088	8.71

**Table 2:** Comparison of the analytical performance of the PEDOT-Tyr/SNGC biosensor with electrochemical biosensors reported in literature.

Biosensor	Sample	Linear Range (µM) and Reference Polyphenol	Limit of Detection (LOD) (µM)	RSD (%)	Ref.
Pt/Polyethersulfone membrane-Laccase	Red wine	2.0–14.0 as (+)-Catechin and Caffeic acid	1.0	<10	[38]
GCE/AuNP-Tyrosinase	White and red wines	2–200 as Caffeic acid	0.66	3.6	[39]
CPE/PBHR-Fc-MWCNT	Wines and teas	0.3–383 as Caffeic acid	0.11	5.2	[40]
CPE/Peroxidase(green bean tissue homogenate)-chitin	White wine	20–200 Caffeic acid	2.0	2.2	[41]
CPE/Tyrosinase	Red wines	20–120	1.6	1.2	[42]
SNGC Nafion/Tyrosinase	Beers	0.6–245 Caffeic acid	1.43	15	[37]
US PES Laccase	Red wines	5–350	0.88	1.9	[43]
Laccase/SNGC	Red and white wines	580–2300	0.71	3.2	[7]
PEDOT-Tyr/SNGC	Wines and beers	10–300 Caffeic acid	4.33	<5	This work
